# The quality of life in patients with multiple sclerosis – Association with depressive symptoms and physical disability: A prospective and observational study

**DOI:** 10.3389/fpsyg.2022.1068421

**Published:** 2023-01-06

**Authors:** Aleksandra Kołtuniuk, Beata Pawlak, Dorota Krówczyńska, Justyna Chojdak-Łukasiewicz

**Affiliations:** ^1^Division of Internal Medicine Nursing, Wroclaw Medical University, Wroclaw, Poland; ^2^Cardinal Stefan Wyszynski Institute of Cardiology, Warsaw, Poland; ^3^Department of Nursing and Obstetrics Collegium Mazovia, Siedlce, Poland; ^4^Department of Neurology, Wroclaw Medical University, Wroclaw, Poland

**Keywords:** nursing, mental health, depression, quality of life, daily activities, multiple sclerosis

## Abstract

**Background:**

Patients with multiple sclerosis (MS) experience disabilities which significantly affect their quality of life (QOL) and mental health. Mood disorders and depressive symptoms are one of the most common psychiatric conditions in MS patients. This study aimed to evaluate the level of QOL in MS patients and to assess the influence of depressive symptoms and physical disability on QOL.

**Methods:**

This prospective and observational study was conducted among 100 MS patients (mean age of 36.23 ± 11.77) recruited from the Lower Silesian Unit of the Polish Association for Multiple Sclerosis. This study used a questionnaire designed by the authors, which contained questions about sociodemographic and clinical data, as well as the following standardized questionnaires: the Activities of Daily Living questionnaire (ADL), the Instrumental Activities of Daily Living questionnaire (IADL), the Expanded Disability Status Scale (EDSS), the Beck Depression Inventory (BDI) and Multiple Sclerosis International Quality of Life Questionnaire (MusiQOL).

**Results:**

The average EDSS score among patients was 3.13 ± 2.38 points. More than half of the respondents (68%) suffered from depression of varying severity. The univariate linear regression models showed that the independent (*p* < 0.05) QOL predictors (total MusiQOL) were as follows: the number of complaints, IADL results, BDI results, EDSS score, higher education, and material status >2000 PLN. In addition, the multiple linear regression model showed that the BDI result was a significant predictor of QOL (*p* < 0.005).

**Conclusion:**

Depressive symptoms significantly affect the QOL of MS patients.

## 1. Introduction

Multiple sclerosis (MS) is a chronic inflammatory, autoimmune and demyelinating disease of the central nervous system characterized by two coexisting processes: inflammation and neurodegeneration (Clarke et al., 2016; Dobson and Giovannoni, 2019). MS can occur at any age, but onset usually occurs around 20 and 40. Females are twice as likely to have MS as males (Logroscino et al., 2018), and young people of working age (Guzik and Kwolek, 2015) are affected. There is an increasing incidence and prevalence of MS in the world. Globally, 2.8 million people suffer from MS; however, its occurrence depends on latitude (Logroscino et al., 2018; Walton et al., 2020). There are more than 700 thousand people (Gitto, 2017) with MS in Europe, with the highest number in Germany – 111,970, and the lowest in Andorra – 96 (Logroscino et al., 2018). In Poland, the approximate prevalence is 120 cases per 100,000 people, which gives an estimated number of 46 thousand patients (Brola et al., 2017). The clinical course of MS is unpredictable and highly variable. Due to the multitude of symptoms resulting from neuronal damage in both the brain and the spinal cord, MS significantly reduces the patients’ functional ability (Huang et al., 2017), and independence (Katsavos et al., 2017), the deterioration of their emotional state (Aşiret et al., 2014), as well as a lower quality of life (QOL) for people affected by the disease compared to the general population (Papuć and Stelmasiak, 2012). Unfortunately, the functional status of MS patients deteriorates over time, leading to various degrees of physical disability (Losy et al., 2016). Research shows that more than 80% of MS patients suffer from varying degrees of disability (Rzepiński et al., 2019), which significantly affects their assessment of their QOL in physical and mental health alike (Mikula et al., 2015).

Neuropsychiatric symptoms frequently occur in MS patients, either as the initial sign complaint prior to a diagnosis or, more commonly, with disease progression. One of the most common is depression (Boeschoten et al., 2017; Henry et al., 2019), a mood disorder that causes persistent sadness and loss of interest. According to the ICD-11, depression is defined by the presence of depressed mood or diminished interest in activities occurring most of the day, nearly every day, for at least 2 weeks, accompanied by other following symptoms: reduced ability to concentrate and sustain attention or marked indecisiveness, beliefs of low self-worth or excessive or inappropriate guilt hopelessness about the future, recurrent thoughts of death or suicidal ideation or evidence of attempted suicide, significantly disrupted sleep or excessive sleep, significant changes in appetite or weight, psychomotor agitation or retardation and reduced energy or fatigue. According to the DSM-5 classification, depression is confirmed with the presence of at least 5 from 8 symptoms during the same 2-week period, and where at least 1 of the symptoms is depressed mood or loss of interest or pleasure. The global prevalence of depression has been still increasing, especially during the COVID-19 pandemic (Vos et al., 2016; Santomauro et al., 2021). The estimated prevalence of depression ranges between 2 and 150/100,000 depending on the specific population. Depression in MS patients is more common than in general population (Papuć and Stelmasiak, 2012). The etiology of depression in MS is unknown. Potential depression causes include genetic, immune-inflammatory, and psychosocial factors. The probable mechanism of depression includes increased proinflammatory cytokines, activation of the hypothalamic–pituitary–adrenal (HPA) axis, and reduction in neurotrophic factors (Arnett et al., 2008).

Depression has a significant impact on the daily function of MS patients. Mood disturbances are an important factor determining QOL in both the mental (Papuć and Stelmasiak, 2012; Barzegar et al., 2018) and the general domain for MS patients (Berrigan et al., 2016; Boogar et al., 2017). Depression significantly affects the course of MS, reduces QQL, and increases the risk of suicidal ideation (Kalb et al., 2019).

QOL was defined in 1995 by the World Health Organization (WHO) as “an individual’s perception of their position in life in the context of the culture and value systems in which they live and concerning their goals, expectations, standards, and concerns. It is a broad-ranging concept affected in a complex way by the person’s physical health, psychological state, level of independence, social relationships, and their relationship to salient features of their environment” [The World Health Organization quality of life assessment (WHOQOL), 1995]. As a chronic disease, MS significantly affects all areas of the patients’ lives. Therefore, assessing the QOL and the impact of the functional and mental state on the QOL of MS patients is as vital for the clinicians as for the patients themselves. It helps to take action to improve and stabilize the patients’ physical and mental condition and enable them to improve the way they perceive their lives (Yalachkov et al., 2019).

Further studies are still in demand to determine the consequences of QOL level in relation to mood disorders and functional status based on standardized research tools. Hence, the study aimed to assess QOL levels in MS patients and the influence of depressive symptoms and physical disability on QOL. We hypothesize that QOL is low among patients with MS and that an increased number of complaints recorded by patients, longer duration of illness, high level of disability, and the presence of depressive symptoms negatively affect the assessment of QOL. Furthermore, we expect that higher education, a better material situation, and support from the environment as defined by status in a relationship positively affect the QOL assessment of patients with MS.

## 2. Materials and methods

### 2.1. Participants

The study was conducted among 100 MS patients with a mean age of 36.23 ± 11.77 years, who were members of the Lower Silesian Unit of the Polish Association for Multiple Sclerosis. Participation in the study was anonymous and voluntary. All patients were informed about the study, and their written consent to participate in the study was taken. The study was carried out in accordance with the guidelines of the Declaration of Helsinki and Good Clinical Practice (World Medical Association, 2013).

The inclusion criteria were as follows: (1) a confirmed diagnosis of MS based on medical records, (2) a stable MS disease without any episodes of relapse within 30 days before the study, (3) being over 18 years old, (4) the Expanded Disability Status Scale (EDSS) score from medical documentation and (5) written informed consent to participate in the study. The exclusion criteria were as follows: (1) participants without a confirmed diagnosis of MS, (2) patients with other neurological disorders (3) patients unable to follow the test instructions, (4) the lack of EDSS score, (5) persons undergoing treatment for depression, (6) patients with other psychiatric disorders and (7) lack of written consent to participate in the study. The degree of physical disability was evaluated in accordance with the Expanded Disability Status Scale (EDSS; Kurtzke, 1983; Walczak et al., 2017). EDSS is a method of quantifying disability in patients with MS and monitoring changes in the level of disability during the progression of the disease. The EDSS scale consists of 20 points between 0 and 10, which specifies intermediate scores between the primary disability levels at an interval of 0.5 points. The scale consists of functional sub-scales to assess the function of the pyramidal system, cerebellum, brainstem, sensory system, sphincters, sight, and higher brain functions; it also includes mobility assessment. The higher the score, the greater the patient’s disability.

The analysis of the material studied showed that the major of respondents were women (82%) with general or technical secondary education (42%), whose gross income per person in the family is between PLN 1001 and PLN 2000 (35%), living in urban areas (80%), married (49%), and who had been ill for less than 5 years (44%). The most frequent complaints of the subjects surveyed included fatigue (81%) and mobility and balance disorders (66%; Table 1).

**Table 1:** Characteristics of the study group.

Variable	Values
Age [years], *mean ± SD*	36.23 ± 11.77
Sex, *n* (%)	
Female	82 (82)
Male	18 (18)
Education, *n* (%)	
Basic or vocational education	21 (21)
Secondary education	42 (42)
Higher education	37 (37)
Place of residence, *n* (%)	
Village	20 (20)
City	80 (80)
Marital status, *n* (%)	
Single	38 (38)
Married	41 (49)
Widowed	2 (2)
Divorced	11 (11)
Material status, *n* (%)	
0–500 PLN	9 (9)
501–1,000 PLN	28 (28)
1,001–2000 PLN	35 (35)
> 2000 PLN	28 (28)
Clinical type of MS, *n* (%)	
RRMS	77 (77)
SPMS	13 (13)
PPMS	4 (4)
PRMS	6 (6)
Disease duration, *n* (%)	
0–5 years	44 (44)
6–10 years	21 (21)
> 10 years	35 (35)
Using DMT, *n* (%)	
Yes	28 (28)
No	72 (72)
EDSS, *mean ± SD (min–max)*	3.13 ± 2.38 (0–10)
ADL (points), *n* (%)	
0–2	14 (14)
3–4	14 (14)
5–6	72 (72)
IADL, *mean ± SD (min–max)*	21.54 ± 3.06 (8–24)
BDI (points), *n* (%)	
0–11	32 (32)
12–26	46 (46)
27–49	18 (18)
50–63	4 (4)
Complains, *n* (%)	
Mobility and balance disorders	66 (66)
Sphincter disorders	49 (49)
Dysphagia	3 (3)
Fatigability	81 (81)
Vision disorders	43 (43)
Hypertonia	47 (47)
Sensory disorders	34 (34)
Speech disorders	14 (14)
Sexual disorders	40 (40)
Mood disorders	26 (26)
Cognitive impairment	30 (30)

RRSM, relapsing–remitting MS; SPMS, secondary progressive MS; PPMS, primary progressive MS; PRMS, progressive-relapsing MS; DMT, disease-modifying therapy; EDSS, Expanded Disability Status Scale; ADL, Activities of Daily Living questionnaire; IADL, Instrumental Activities of Daily Living questionnaire; BDI, Beck Depression Inventory.

### 2.2. Study design

A prospective and descriptive design with a questionnaire survey was used. The study was conducted between January 2017 and May 2017. The study follows the Strengthening the Reporting of Observational Studies in Epidemiology (STROBE) recommendations for reporting of observational studies. The research project was approved by the Bioethics Committee of Wroclaw Medical University (no. KB–176/2017).

### 2.3. Procedure

The respondents were qualified to participate in this study based on the inclusion and exclusion criteria during a meeting for members of the Lower Silesian Unit of the Polish Association for Multiple Sclerosis. Afterward, they received traditional self-administered pencil-and-paper questionnaires designed to be completed in approximately 30 min. Researchers also gained access to the full medical records of the patients. A total of 115 surveys were completed, but only 100 were correctly completed.

### 2.4. Questionnaires

In the study, the diagnostic survey method was used, along with the author’s questionnaire, as well as the following standardized questionnaires: the Activities of Daily Living questionnaire (ADL; Katz et al., 1963; Borowicz, 2011), the Instrumental Activities of Daily Living questionnaire (IADL; Lawton, 1971; Borowicz, 2011), the Beck Depression Inventory (BDI; Beck et al., 1961; Paradowski and Jernajczyk, 1977) and the Multiple Sclerosis International Quality of Life Questionnaire (MusiQOL; Jamroz-Wiśniewska et al., 2011). Author’s Questionnaire (AQ) included a survey designed by the authors, which included questions about sociodemographic data (i.e., sex, age, place of residence, marital status, education, material status) and clinical data (i.e., the time since the diagnosis and the occurrence of symptoms). The Activities of Daily Living questionnaire (ADL) assess the respondent’s capability of handling their daily activities. The questionnaire consists of 6 questions (actions). The patients must determine if they can carry out the above activities independently, with 2 answers to choose from – “yes,” for which 1 point is given, and “no,” for which 0 points are given. The final result is the number of activities that the patient can perform independently. Persons with scores of 5–6 are non-disabled, whereas those with 3–4 are moderately disabled and those with a score equal to or lower than 2 are significantly disabled. The Instrumental Activities of Daily Living questionnaire (IADL) assess the patient’s ability to perform complex everyday activities. The IADL scores range between 8 and 24 points. The higher the result, the more independent the patient is. In the case of IADL, there are no norms that would make it possible to determine the score range corresponding to either high or low autonomy. The total number of points obtained is relevant only to a specific patient. The decrease in this number over time indicates a deterioration of the patient’s general condition. Reliability of this questionnaire was established at 0.85 (Graf, 2008). Beck Depression Inventory (BDI) is a self-report measure of depression severity. Scores ranging from 0 to 11 indicate a lack of depression; 12–26 points – mild depression; 27–49 points – moderately severe depression; and 50–63 – very severe depression. The BDI demonstrates high internal consistency, with alpha coefficients of 0.86 and 0.81 for psychiatric and non-psychiatric populations, respectively (Beck et al., 1988). The Multiple Sclerosis International Quality of Life Questionnaire (MusiQOL) is a research tool aimed at assessing the QOL of people with MS. It contains 31 questions about the patient’s life during the last 4 weeks, with the following verbal answers: never, rarely, sometimes, often, always, not applicable. The MusiQOL questionnaire makes it possible to assess the QOL of MS patients in 10 domains: ADL – activities of daily living, PWB – psychological well-being, RFr – relationships with friends, SPT – symptoms, RFa –relationships with family, RHCS – relationship with the healthcare system, SSL – sentimental and sexual life, COP – coping, REJ – rejection, Total – overall QOL. The QOL in each domain is expressed by a number ranging from 0 to 100—the higher the number, the better the QOL. No norms exist in the case of MusiQOL, and as such, it is impossible to say whether the respondents’ results indicate high or low QOL; one can only compare the individual domains with each other to identify the areas of high and low QOL. The dimensions of the scale exhibited high internal consistency (Cronbach’s alpha between 0.67 and 0.90 for the Polish version).

### 2.5. Statistical analysis

The analysis of quantitative variables (i.e., numeric ones) was carried out by calculating the mean, standard deviation, median, and quartiles, as well as the minimum and maximum. The analysis of quality variables (i.e., non-numeric ones) was carried out by calculating the number and percentage of occurrences of each value. The comparison of the quantitative variable values in two groups was made using Student’s t-test (when the variable had a normal distribution in the groups analyzed) and Mann–Whitney’s test (when it had no normal distribution). The comparison of the quantitative variable values in three or more groups was performed using the analysis of variance (ANOVA – when the variable had a normal distribution in the groups analyzed) and the Kruskal-Wallis test (when it had no normal distribution). If statistically significant differences were observed, a *post hoc* analysis using Fisher’s least significant difference (LSD) test for normal distribution and Dunn’s test for non-normal distribution was performed to determine which specific groups differ from one another. Linear regressions were used to analyze the impact of potential predictors on a quantitative variable. Regression parameters with 95% confidence intervals were shown. Variables to include in multiple regression were selected based of their significance in simple regressions. Variables with the lowest *p*-values were chosen so that SPV (Subjects Per Variable) index was at least 10. The significance level for all statistical tests was set to 0.05. R 4.2.2. was used for computations.

## 3. Results

### 3.1. Disability level

Analysis of the EDSS showed that the patients’ average score was 3.13 (*SD* = 2.38) and ranged from 0 to 9. 72% of the survey participants were able-bodied (5–6 points in the ADL scale), and 14% were moderately disabled (3–4 points in the ADL scale), with another 14% being significantly disabled (0–2 points in the ADL scale). The average number of points obtained by the respondents in the IADL questionnaire was 21.54 (*SD* = 3.06) and ranged from 8 to 24. Only one person got the lowest possible score (8 points), while 39% got the highest (24 points). It was shown that the number of complaints is among the factors associated with the outcome of an ADL – the higher the number of complaints, the higher the degree of the patient’s disability (*r =* −0.231*, p* = 0.021). On the other hand, the IADL results are associated with the patient’s age (*r =* −0.382*, p* < 0.001), gross income per family member (patients with income above PLN 2000 were significantly more capable when undertaking complex everyday activities than the rest of the patients, *p* = 0.004), disease duration (patients who had been suffering from the disease for less than 5 years were significantly more capable, *p* < 0.001), number of complaints (the greater the number of complaints, the higher the degree of the patient’s disability, *r* = −0.418*, p* < 0.001). Additionally, it was shown that the EDSS score is associated with the patient’s age (*r* = 0.625, *p* < 0.001), marital status (single achieved significantly lower EDSS results than other patients, which means a lower degree of disability, *p* = 0.001), gross income per family member (patients with an income between PLN 1001 and 2000 are characterized by a significantly a higher degree of disability than patients with income above PLN 2000), disease duration (patients suffering from the disease for longer than 10 years were characterized by a higher degree of disability, *p* < 0.001), number of complaints (the greater the number of complaints, the higher the degree of the patient’s disability, *r* = −0.447, *p* < 0.001).

### 3.2. Depressive symptoms

Analysis of the data obtained using the depressive symptom severity assessment questionnaire showed that more than half of the respondents (68%) suffered from depression at varying degrees of severity (46% of the respondents had symptoms of mild depression while 22% of them had symptoms of moderate and severe depression). It was also shown that the severity of symptoms might indicate that depression is associated with age (*r* = −0.236*, p* < 0.001). Furthermore, patients with primary and vocational education were characterized by a higher severity of depression symptoms than patients with a university education (*p* = 0.036); additionally, those who reported a higher number of complaints exhibited a higher severity of depression symptoms as well (*r* = 0.369*, p* < 0.001).

### 3.3. Quality of life

The average number of points obtained by respondents in the MusiQOL questionnaire was 58.21 (*SD* = 18.06) out of 100, ranging from 21.53 to 96.53. The respondents assessed their QOL to be the highest in rejection, relationships with family, and symptoms domains. In contrast, these assessments were the lowest in the relationship with the healthcare system, psychological well-being, and coping domains. Detailed data are presented in Table 2.

**Table 2:** Assessment of the overall quality of life and the individual domains of the MusiQOL scale.

Domain	*N*	*M*	*SD*	*Me*	*Min*	*Max*	*Q1*	*Q3*
Total QoL	86	58.21	18.06	57.64	21.53	96.53	43.79	69.21
ADL	97 *	56.4	24.31	53.57	7.14	100	37.5	78.12
PWB	100	51.5	25.41	50.0	0	100	31.25	68.75
RFr	95 *	57.19	26.0	58.33	0	100	33.33	75
SPT	99 *	63.01	21.84	62.5	12.5	100	50	81.25
RFa	100	67.25	30.82	75	0	100	41.67	100
RHSC	98 *	49.45	28.28	50	0	100	25	75
SSL	97 *	53.61	32.12	50	0	100	25	87.5
COP	99 *	52.4	28.78	50	0	100	25	75
REJ	98 *	74.62	27.07	75	0	100	50	100

* Some respondents chose “not applicable” in too many questions to calculate the total score. N, number of participants; M, mean; SD, standard deviation; Me, median; Min, minimum; Max, maximum; Q1, lower quartile; Q3, upper quartile; QoL, quality of life; ADL, activities of daily living; PWB, psychological well-being; RFr, relationships with friends; SPT, symptoms; RFa, relationships with family; RHCS, relationship with the healthcare system; SSL, sentimental and sexual life; COP, coping with the disease; REJ, rejection.

The data analysis showed that men had a higher QOL in psychological well-being and symptoms compared to women (*p* < 0.05). For MS patients, the QOL in the coping domain (*r* = 0.302*, p* = 0.002) and in the activities of daily living domain (*r* = −0.393*, p* < 0.001) was associated with age. Patients with a university education rated their QOL in the relationships with family (*p* = 0.031), coping (*p* = 0.042), and rejection (*p* = 0.009) domains significantly higher than other subjects. City inhabitants were characterized by a higher QOL in the coping domain compared to people from rural areas (*p* < 0.05). While single achieved significantly higher scores in the activities of daily living domain of the MusiQOL scale compared to the widowed and divorced people (*p* = 0.039), the widowed and divorced patients achieved significantly higher scores in the coping domain compared to other patients, which means that they found it easier to come to terms with the disease (*p* = 0.042). Compared to patients with an income above PLN 2000, patients with an income between PLN 1001 and PLN 2000 were characterized by a significantly lower QOL in the activities of daily living domain (*p* = 0.042). Patients who have been ill for less than 5 years enjoyed a higher QOL in the activities of daily living (*p* < 0.001) and rejection (*p* = 0.044) domains. The number of complaints was associated with the scores in nine out of the 10 scales of the MusiQOL questionnaire (*p* < 0.05; coping – being the only exception).

**Figure 1:**
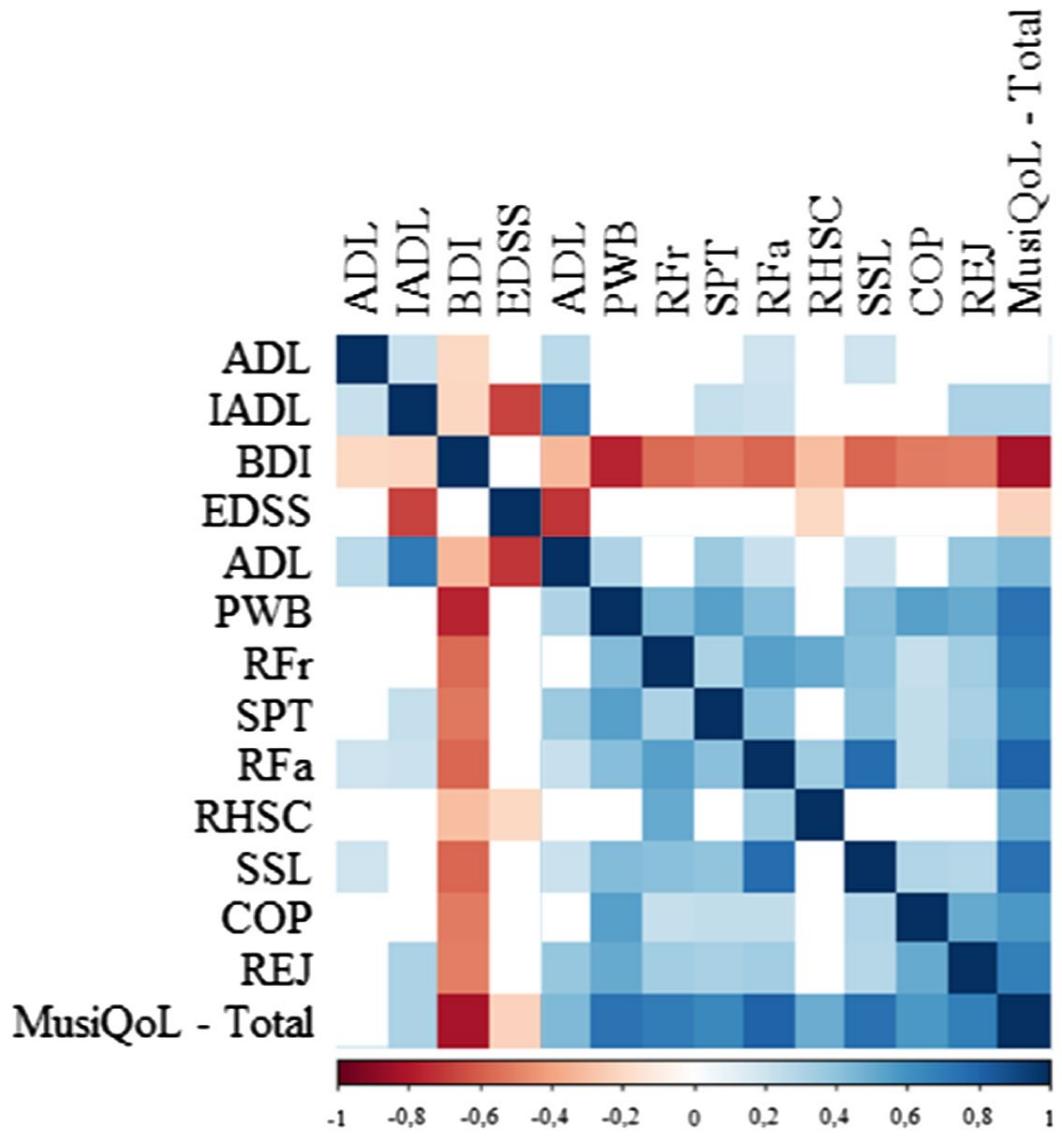
Spearman rank correlation among the variables used.

The Spearman’s rank correlation coefficient is presented in Figure 1 and the detailed statistical analysis (value of *p*) is presented in Supplementary Tables 1–7.

The univariate linear regression model showed that the independent (*p* < 0.05) QOL predictors (total MusiQoL) were as follows: the number of complaints, IADL results, BDI results, EDSS score, higher education, and material status >2000 PLN. The multiple linear regression model showed that the independent (*p* < 0.05) QOL predictor is only the BDI score (Table 3).

**Table 3:** Results of linear regression model indicating independent predictors of the QOL.

Variables		*Univariate regression*				*Multiple regression*			
		*B*	*95%CI*		*p*	*B*	*95%CI*		*p*
Age		0.081	−0.263	0.425	0.645				
Number of complains		−3.684	−5.313	−2.055	<0.001 *	−0.46	−1.72	0.801	0.477
ADL		1.342	−0.845	3.529	0.233				
IADL		1.449	0.268	2.631	0.018 *	0.207	−0.764	1.178	0.677
BDI		−1.436	−1.657	−1.215	<0.001 *	−1.328	−1.577	−1.08	<0.001 *
EDSS		−1.723	−3.304	−0.143	0.036 *	−0.887	−2.258	0.484	0.209
Sex	Female	ref.							
	Male	2.845	−6.572	12.262	0.555				
Education	Basic or vocational	ref.				ref.			
	Secondary	1.766	−7.93	11.462	0.722				
	Higher	10.707	0.786	20.628	0.037 *	2.559	−2.316	7.433	0.307
Place of residence	City	ref.							
	Village	−4.795	−13.991	4.402	0.31				
Marital status	Single	ref.				ref.			
	Married	3.287	−4.813	11.387	0.429				
	Widowed and divorced	11.72	−1.507	24.946	0.086	3.273	−4.326	10.872	0.401
Material status	< 1,000 PLN	ref.				ref.			
	501–1,000 PLN	2.821	−6.198	11.84	0.542				
	> 2000 PLN	10.457	1.317	19.597	0.028 *	0.048	−5.393	5.489	0.986
Disease duration	< 5 years	ref.							
	5–10 years	−2.469	−12.318	7.38	0.625				
	> 10 years	−3.476	−12.354	5.401	0.445				

* Statistically significant. ADL, Activities of Daily Living questionnaire; IADL, Instrumental Activities of Daily Living questionnaire; BDI, Beck Depression Inventory; QoL, quality of life.

## 4. Discussion

### 4.1. Quality of life assessment

The first aim of this study was to assess the level of QOL in MS patients. Recent studies (Farran et al., 2020; Al Jumah et al., 2021; Mitsikostas et al., 2021) indicate that MS patients, regardless of where they live, assess their QOL positively. These findings are in line with our results. This state of affairs is most likely related to the increased availability of MS treatment.

Another finding worthy of further investigation is the significantly worse impact of MS on women’s QOL. The study showed that the female gender was associated with assessing the QOL in the field of mental well-being (PWB) and disease symptoms (SPT). In the MusiQOL scale, men rated these two areas significantly higher than women. The study conducted in Oman showed that the psychological well-being and coping domains of MusiQoL questionnaires are significantly reduced in females compared to males (Natarajan et al., 2021). Miller and Dishon (Miller and Dishon, 2006) suggest that women with MS, compared to men, have more significant difficulties due to their symptoms, which have a major impact on well-being and daily functioning. On the other hand, the analysis of the data obtained by Barzegar et al. (Barzegar et al., 2018) showed that women with MS achieved higher scores in the QOL assessment. The authors claim that women have better mechanisms for adapting to difficulties, so they do not affect the evaluation of their QOL to such an extent. However, many authors (Jamroz-Wiśniewska et al., 2011; Mikula et al., 2015; Brola et al., 2016) state that no significant statistical difference between the genders in the QOL assessment was observed in their research.

The impact of age on the mental functioning assessment can seem puzzling. However, the analysis showed that the older the patient is, the higher the QOL in the PWB and COP subscales, relating to mental well-being and coping with the disease. Similarly, other studies (Buchanan et al., 2008; Jones and Amtmann, 2015) observed a positive correlation between age and assessing one’s mental well-being. People suffering from chronic, long-term diseases must continuously adapt to changes in their condition caused by new and existing, steadily worsening symptoms. The most vital thing here is to find the best coping strategies, which are often developed throughout one’s life (Pierobon et al., 2011). Therefore, thanks to their thoroughly developed adaptation methods, older patients are the most likely to cope better with the disease and rate their mental health quality higher.

Disease duration is yet another factor that lowers the QOL. It mainly affects the evaluation of the physical sphere. As the disease progresses, the patients become more dependent on others, which lowers their QOL. Other authors obtained similar results in their studies (Tadić and Dajić, 2013; Snarska et al., 2016; Yalachkov et al., 2019), showing that patients with MS that lasted longer rated both their degree of disability and QOL at a lower level. However, in some works (Jamroz-Wiśniewska et al., 2007; Sehanovic et al., 2011), there was no correlation between illness duration and the degree of disability and the QOL assessment. Such differences may result from the sample size; e.g., Sehanovic et al. (2011) tested only a group of 50 MS patients; or the fact that different varieties of MS exist (Jamroz-Wiśniewska et al., 2007; Humańska et al., 2013). The most common one is relapsing–remitting MS, which changes into progressive MS (Miller and Leary, 2007), but there are also mild forms, in the case of which the disease does not necessarily develop over time (Humańska et al., 2013).

In the present study, a higher QOL characterized people with a university education. Research by other authors also confirms this relationship (Jamroz-Wiśniewska et al., 2011; Tadić and Dajić, 2013; Šabanagić-Hajrić and Alajbegović, 2015; Snarska et al., 2016; Yalachkov et al., 2019). Research by Snarska et al. (2016) showed that people with a university education enjoy a higher QOL, especially in the social domain. Other researchers proved that this relationship applies to virtually every QOL area (Jamroz-Wiśniewska et al., 2007, 2011; Tadić and Dajić, 2013). People who are better educated have a greater sense of security and more opportunities to develop and implement their plans. Besides, their knowledge may enable them to assess their health, notice any abnormalities quicker, and begin treatment faster.

Research shows (Buchanan et al., 2008; Papuć and Stelmasiak, 2012; Schmidt and Jöstingmeyer, 2019) that the QOL assessment is related to the presence of friends and loved ones. They provide a support system and a sense of security, and facilitate adaptation to the disease’s limitations, thus improving the QOL assessment. In our study, the respondents rated their QOL in family relations and societal functioning as the highest.

The way the place of residence affects the QOL assessment in MS patients is a contentious issue. A study by Buchanan et al. (2008) and Jamroz-Wiśniewska et al. (2007, 2011) shows that city inhabitants enjoy a higher QOL. The analysis of the author’s research material showed that the place of residence does not significantly affect most domains. Differentiating patients from urban and rural areas is the value of COP from the MusiQOL questionnaire, which determines the extent to which the patient has come to terms with the disease. In this variable, city inhabitants scored higher than patients living in rural areas. This is most likely because people living in urban enjoy better access to doctors of various specialties and new medicinal treatment solutions, and other forms of therapy, and as such, they have a better chance of receiving the help necessary to overcome difficulties (Jamroz-Wiśniewska et al., 2007, 2011). In towns and cities, MS patients can join support groups and participate in various activities – with transportation often being provided. All these activities help patients to remain as active and efficient as possible.

The patient’s financial situation is another vital factor affecting their state. The better the patient’s financial situation, the higher they rate their health and ability to function independently. Patients whose income allows them to live comfortably enjoy a higher QOL (Abdullah and Badr, 2018). The author’s study reflects this, as well. People with a higher income rated their QOL in the physical and social areas higher. In addition, more affluent patients have better access to different medical specialists and treatment methods, which improves their health condition and the range of activities they can perform independently.

### 4.2. Functional status and its impact on the quality of life

The analysis of the research material showed that age is associated with the patient’s condition. Their fitness level and ability to perform complex everyday activities deteriorate with age, and research by other authors confirms this as well (Papuć and Stelmasiak, 2012; Łabuz-Roszak et al., 2013; Snarska et al., 2016). It was also shown that a higher number of points obtained in the EDSS scale correlates with a lower number of points obtained in the IADL scale and the QOL in the ADL field. This means that a higher degree of the patient’s disability is accompanied by decreased independence and ability to take care of themselves. As a result, patients have a negative view of the QOL lives. Other authors (Sehanovic et al., 2011; Papuć and Stelmasiak, 2012; Tadić and Dajić, 2013; Mikula et al., 2015; Berrigan et al., 2016; Brola et al., 2016; Ochoa-Morales et al., 2019; Schmidt and Jöstingmeyer, 2019) obtained similar results – patients with a higher degree of disability were characterized by a lower QOL assessment, especially in terms of their physical functioning.

### 4.3. Depression and its impact on the quality of life

Psychiatric symptoms are common in MS patients, with a point prevalence of depression among clinic patients of 15–30% and a lifetime prevalence of 40–60%. Therefore, it is estimated that depression symptoms occur in about 38% of them (Hernández-Ledesma et al., 2018; Henry et al., 2019). However, in the studies by Winiecka et al. (2016) and the author’s study, more than half of the respondents exhibited depression at varying degrees of severity. Winiecka et al. (2016) showed that depression symptoms intensify with age; however, an inverse relationship was observed in the author’s study. It may be related to developing specific mechanisms that can adapt to the disease over time.

Barzegar et al. (2018) and Shamsaei et al. (2015) showed that men suffering from MS experience more severe depression symptoms than women; however, this was not confirmed in the author’s study. However, the hereby confirms a correlation between the level of education and the severity of depression symptoms—individuals with a university education experience fewer symptoms of depression (Winiecka et al., 2016; Hanna and Strober, 2020).

Although the previous research (Winiecka et al., 2016; Schmidt and Jöstingmeyer, 2019) showed that MS patients who are physically and professionally active exhibit a lower level of depression, this study did not confirm this relationship.

When diagnosing depression in patients with MS, one should first look for symptoms, i.e., sadness, guilt, disappointment, feelings of failure, and pessimism, accompanied by appetite or weight changes. Then, suppose the loss of interest, crying, dissatisfaction, irritability, and self-criticism are also found. In that case, additional investigations should be carried out to determine whether these symptoms exceed what would be expected in MS, as many MS symptoms are also characteristic of depression (Strober and Arnett, 2010).

A correct diagnosis of depression in MS patients is crucial and previous studies showed that depression is a strong predictor of MS patients’ QOL (Berrigan et al., 2016; Fricska-Nagy et al., 2016; Boogar et al., 2017; Hernández-Ledesma et al., 2018; Ochoa-Morales et al., 2019; Schmidt and Jöstingmeyer, 2019; Yalachkov et al., 2019), which it was proved in our study. Since depression can exacerbate clinically troublesome problems such as pain, fatigue, anxiety, and cognitive disorders (Feinstein et al., 2014), MS patients’ mental health must be systematically assessed, and effective treatment must be implemented. Studies showed that patients who have undergone depression treatment rated their QOL higher (Hofmann et al., 2017) than before. Moreover, Mohr et al. (1997) suggested that treating depression may help MS patients to adhere to therapeutic recommendations; this was partially confirmed by Tarrants et al. (2011), who demonstrated that at least a 6-month-long antidepressant treatment is associated with better adherence to the DMT recommendations.

**Figure 2:**
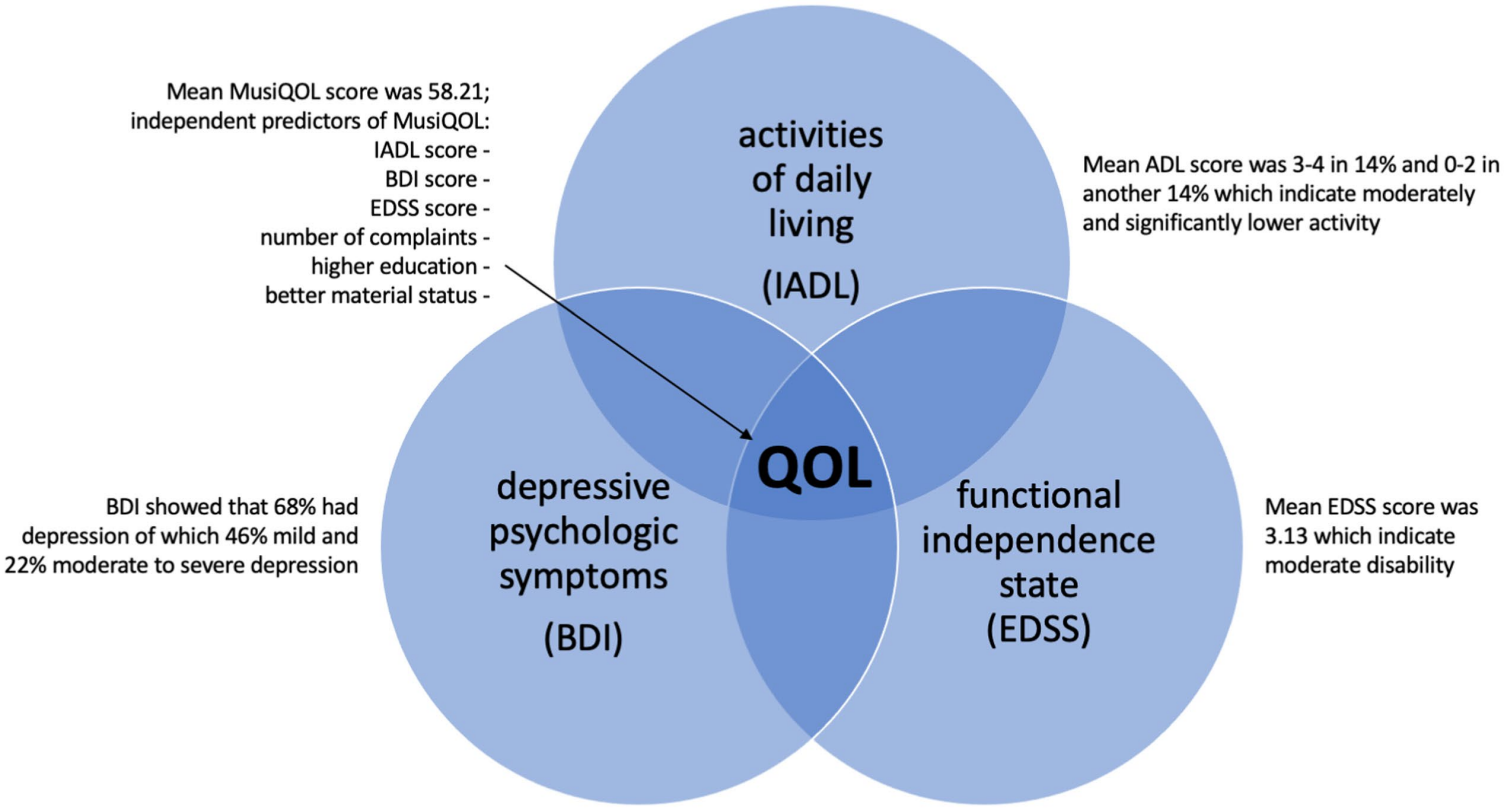
Summary of the relationship in the presented model based on our findings.

In terms of clinical relevance, the research showed that depressive symptoms affect the QOL of MS patients. Professional team members should be capable of recognizing the depressive symptoms, and these symptoms should be taken into account when planning patient care. However, standardized screening tools are used infrequently because of the lack of support staff (Tornatore et al., 2022). Screening for depression should be an ongoing part of the therapeutic process, especially when patients are undergoing DMT treatment because patients characterized by depressive symptoms are less adhere to therapeutic recommendations (Kołtuniuk and Rosińczuk, 2021). Figure 2 presents the summary of the relationship in the presented model based on our findings.

## 5. Limitation

This study has several potential limitations. Firstly, the study participants were enrolled in a meeting of the association for MS patients, where they received additional informational and emotional support. Therefore, this is not a representative group, and the results should not be generalized for all MS patients. Secondly, the group of respondents involved in the study should be more extensive and consist of a larger patient group subjected to DMT treatment. Thirdly, this study does not include a control group, which should also be considered in future studies. Furthermore, all research tools used are of the self-report type, and there is a risk of self-report bias. Moreover, the questionnaire used to assess depression was straightforward; therefore, the Depression Assessment Questionnaire by Łojek et al. (2015) should be considered in future studies.

## 6. Conclusion

Patients with MS assess their QoL positively. Mood disorders, i.e., depression, significantly affect the QOL of MS patients. Although a relationship between the patient’s functional status and their QOL was observed, the multiple regression model did not confirm the impact of the level of physical disability on the total MusiQOL.

## Data availability statement

The raw data supporting the conclusions of this article will be made available by the authors, without undue reservation.

## Ethics statement

The studies involving human participants were reviewed and approved by Bioethics Committee of Wroclaw Medical University (no. KB–176/2017). The patients/participants provided their written informed consent to participate in this study.

## Author contributions

All authors of this manuscript meet the authorship criteria according to the latest guidelines of the International Committee of Medical Journal Editors (ICMJE). AK and JC-Ł: conceptualization, formal analysis, writing – original draft, supervision, and project administration. AK, BP, DK, and JC-Ł: methodology and writing – review and editing. AK and BP: investigation. AK, BP, and JC-Ł: data curation. AK and DK: visualization and funding acquisition. AK, BP, and DK: software. All authors contributed to the article and approved the submitted version.

## Funding

This work was supported by the Ministry of Health subventions according to number of SUBZ.E250.22.095 from the IT Simple system of the Wroclaw Medical University.

## Conflict of interest

The authors declare that the research was conducted in the absence of any commercial or financial relationships that could be construed as a potential conflict of interest.

## Publisher’s note

All claims expressed in this article are solely those of the authors and do not necessarily represent those of their affiliated organizations, or those of the publisher, the editors and the reviewers. Any product that may be evaluated in this article, or claim that may be made by its manufacturer, is not guaranteed or endorsed by the publisher.
