# Supplementary material for: The quality of life in patients with multiple sclerosis – Association with depressive symptoms and physical disability: A prospective and observational study
Source: Front Psychol. 2023 Jan 6;13:1068421. doi: 10.3389/fpsyg.2022.1068421 (PMC9853525; doi:10.3389/fpsyg.2022.1068421)
Supplement: Supplementary file 1 [file Table_1.DOCX]

**Supplementary Table 1.** Comparison of questionnaire results by gender and place of residence.

|  | | Female (*n*=82) | Male  (*n*=18) | *p** | City (*n*=80) | Village (*n*=20) | *p** |
| --- | --- | --- | --- | --- | --- | --- | --- |
|  |  | *M (SD)* | *M (SD)* |  | *M (SD)* | *M (SD)* |  |
| ADL | | 4.8 (1.6) | 4.3 (2.2) | 0.461 | 4.6 (1.8) | 5 (1.5) | 0.241 |
| IADL | | 21.8 (2.5) | 20.2 (4.7) | 0.359 | 21.6 (3.1) | 21.2 (3.1) | 0.504 |
| BDI | | 17.1 (10.6) | 16.7 (11.9) | 0.9 | 16.2 (10.2) | 20 (12.5) | 0.222 |
| EDSS | | 3 (2.3) | 3.7 (2.7) | 0.365 | 3.2 (2.4) | 2.9 (2.3) | 0.674 |
| MusiQOL | ADL | 57.3 (23.2) | 52.3 (28.9) | 0.388 | 55.4 (24) | 60.1 (25.8) | 0.509 |
|  | PWB | 48.2 (24.6) | 66.3 (24.4) | 0.008 | 53 (25.3) | 45.3 (25.6) | 0.252 |
|  | RFr | 58 (25.4) | 53.7 (28.9) | 0.586 | 58 (25.7) | 53.9 (27.6) | 0.553 |
|  | SPT | 60.7 (21.6) | 73.3 (20.7) | 0.02 | 63 (21.7) | 63 (23) | 0.906 |
|  | RFa | 67.3 (30.7) | 67.1 (32.3) | 0.993 | 67.5 (32.2) | 66.2 (25.1) | 0.562 |
|  | RHSC | 52.2 (27.4) | 37 (29.6) | 0.051 | 50.5 (27.8) | 45.2 (30.6) | 0.476 |
|  | SSL | 53.5 (31.6) | 54.2 (35.1) | 0.87 | 52.9 (31.5) | 56.2 (35.2) | 0.676 |
|  | COP | 50 (27.7) | 63.2 (31.6) | 0.088 | 56.8 (28.4) | 35 (23.5) | 0.003 |
|  | REJ | 74.1 (27.3) | 77.1 (26.5) | 0.655 | 77.4 (25.2) | 63.8 (31.9) | 0.07 |
|  | Total | 57.6 (17.9) | 60.5 (18.9) | 0.51 | 59.3 (18) | 54.5 (18.4) | 0.252 |

* Mann-Whitney U test (due to non-normality of distribution).

M, mean; SD, standard deviation; N, number of participants; p, statistical significance; ADL, activities of daily living; IADL, Instrumental Activities of Daily Living questionnaire; BDI, Beck Depression Inventory; EDSS, Expanded Disability Status Scale; PWB, psychological well-being; RFr, relationships with friends; SPT, symptoms; RFa, relationships with family; RHCS, relationship with the healthcare system; SSL, sentimental and sexual life; COP, coping with the disease; REJ, rejection.

**Supplementary Table 2.** Correlation with age, number of complaints and results of questionnaires.

|  | Correlation with age | | Correlation with number of complaints | |
| --- | --- | --- | --- | --- |
|  | *β* | *p* | *β* | *p* |
| ADL | -0.142 | 0.159 | -0.231 | 0.021 |
| IADL | -0.382 | <0.001 | -0.418 | <0.001 |
| BDI | -0.236 | 0.018 | 0.369 | <0.001 |
| EDSS | 0.625 | <0.001 | 0.447 | <0.001 |
| ADL | -0.393 | <0.001 | -0.552 | <0.001 |
| PWB | 0.245 | 0.014 | -0.269 | 0.007 |
| RFr | 0.042 | 0.689 | -0.226 | 0.027 |
| SPT | 0.033 | 0.746 | -0.318 | 0.001 |
| RFa | 0.01 | 0.923 | -0.274 | 0.006 |
| RHSC | 0.005 | 0.958 | -0.292 | 0.004 |
| SSL | 0.077 | 0.456 | -0.383 | <0.001 |
| COP | 0.302 | 0.002 | -0.042 | 0.681 |
| REJ | 0.083 | 0.414 | -0.22 | 0.029 |
| MusiQOL - Total | 0.087 | 0.426 | -0.439 | <0.001 |

p, statistical significance; ADL, activities of daily living; IADL, Instrumental Activities of Daily Living questionnaire; BDI, Beck Depression Inventory; EDSS, Expanded Disability Status Scale; PWB, psychological well-being; RFr, relationships with friends; SPT, symptoms; RFa, relationships with family; RHCS, relationship with the healthcare system; SSL, sentimental and sexual life; COP, coping with the disease; REJ, rejection.

**Supplementary Table 3.** Comparison of questionnaire results by education.

|  | | Basic or vocational education (*n*=21) | Secondary education (*n*=42) | Higher  education (*n*=37) | *p* * |
| --- | --- | --- | --- | --- | --- |
|  |  | *M (SD)* | *M (SD)* | *M (SD)* |  |
| ADL | | 4.1 (2.1) | 4.9 (1.6) | 4.8 (1.7) | 0.198 |
| IADL | | 20.7 (2.9) | 21.7 (3.2) | 21.8 (3) | 0.108 |
| BDI | | 20.9 (11.9) | 17.4 (8.9) | 14.4 (11.5) | 0.036 a |
| EDSS | | 3.5 (2.3) | 3.2 (2.5) | 2.9 (2.3) | 0.662 |
| MusiQOL | ADL | 48.8 (18.8) | 55.7 (25.8) | 61.6 (24.7) | 0.131 |
|  | PWB | 49.7 (24.2) | 48.5 (21.8) | 55.9 (29.6) | 0.295 |
|  | RFr | 55 (26.5) | 55.4 (23) | 60.5 (29.2) | 0.618 |
|  | SPT | 63.6 (22.7) | 61.4 (18.5) | 64.4 (25) | 0.678 |
|  | RFa | 61.1 (30.4) | 62.9 (30.6) | 75.7 (30.2) | 0.031 b |
|  | RHSC | 47.1 (26.3) | 52.8 (25.9) | 47 (31.9) | 0.552 |
|  | SSL | 46.4 (36) | 49.7 (28.4) | 62.9 (32.8) | 0.126 |
|  | COP | 43.5 (31) | 48.8 (24.8) | 61.8 (29.9) | 0.042 c |
|  | REJ | 66.7 (28.3) | 71 (24.9) | 83.3 (27.1) | 0.009 b |
|  | Total | 53.6 (15.3) | 55.4 (15) | 64.3 (21.4) | 0.073 |

* Kruskal-Wallis test (due to non-normality of distribution) + post-hoc analysis (Dunn’s test).

a Basic or vocational education > Higher education;

b Basic or vocational < Higher education. Secondary education < Higher education;

c Basic or vocational education < Higher education.

M, mean; SD, standard deviation; N, number of participants; p, statistical significance; ADL, activities of daily living; IADL, Instrumental Activities of Daily Living questionnaire; BDI, Beck Depression Inventory; EDSS, Expanded Disability Status Scale; PWB, psychological well-being; RFr, relationships with friends; SPT, symptoms; RFa, relationships with family; RHCS, relationship with the healthcare system; SSL, sentimental and sexual life; COP, coping with the disease; REJ, rejection.

**Supplementary Table 4.** Comparison of questionnaire results by marital status.

|  | | Single (n=38) | Married (*n*=41) | Others (*n*=13) | *p* * |
| --- | --- | --- | --- | --- | --- |
|  |  | *M (SD)* | *M (SD)* | *M (SD)* |  |
| ADL | | 4.7 (1.8) | 4.9 (1.7) | 4.1 (2) | 0.282 |
| IADL | | 22 (2.9) | 21.4 (3.1) | 20.7 (3.4) | 0.195 |
| BDI | | 18.7 (11.1) | 17.1 (10.8) | 11.8 (8.4) | 0.117 |
| EDSS | | 2.1 (2.2) | 3.5 (2.2) | 4.7 (2.4) | 0.001 a |
| MusiQOL | ADL | 63.9 (22.5) | 53.5 (23.7) | 44.8 (26.7) | 0.039 b |
|  | PWB | 47.4 (26.7) | 51.4 (24.4) | 63.9 (22.8) | 0.115 |
|  | RFr | 57.4 (28.1) | 53.4 (24.5) | 69.9 (22.7) | 0.158 |
|  | SPT | 61.3 (23.6) | 65.9 (19.9) | 56.8 (23.8) | 0.421 |
|  | RFa | 60.1 (33.1) | 69.9 (30.3) | 78.2 (21.7) | 0.202 |
|  | RHSC | 49.8 (26.7) | 46.9 (28.1) | 57.7 (33.9) | 0.578 |
|  | SSL | 45.9 (37.7) | 56.1 (27.8) | 68.2 (24) | 0.077 |
|  | COP | 48.4 (29.2) | 50.5 (28.7) | 71.2 (21.3) | 0.042 c |
|  | REJ | 70.6 (30.4) | 74.2 (25.6) | 87.5 (19.1) | 0.156 |
|  | Total | 55.3 (20.7) | 58.6 (15.9) | 67 (16.2) | 0.142 |

* Kruskal-Wallis test (due to non-normality of distribution) + post-hoc analysis (Dunn’s test).

a Single < Others, Single < Married;

b Single > Others;

c Single < Others, Married < Others.

M, mean; SD, standard deviation; N, number of participants; p, statistical significance; ADL, activities of daily living; IADL, Instrumental Activities of Daily Living questionnaire; BDI, Beck Depression Inventory; EDSS, Expanded Disability Status Scale; PWB, psychological well-being; RFr, relationships with friends; SPT, symptoms; RFa, relationships with family; RHCS, relationship with the healthcare system; SSL, sentimental and sexual life; COP, coping with the disease; REJ, rejection.

**Supplementary Table 5.** Comparison of questionnaire results by gross income per family member (PLN).

|  | | <1000 PLN (*n*=37) | 1001-2000 PLN (*n=*35) | >2000 PLN (*n*=28) | *p* * |
| --- | --- | --- | --- | --- | --- |
|  |  | *M (SD)* | *M (SD)* | *M (SD)* |  |
| ADL | | 4.7 (1.5) | 4.9 (1.5) | 4.4 (2.3) | 0.897 |
| IADL | | 21 (3.1) | 21 (3.3) | 22.9 (2.2) | 0.004 a |
| BDI | | 18.2 (9.7) | 17.7 (12.1) | 14.6 (10.3) | 0.293 |
| EDSS | | 3.2 (2.3) | 3.9 (2.3) | 2.1 (2.4) | 0.008 b |
| MusiQOL | ADL | 54.7 (22.7) | 50.5 (23.3) | 66.7 (25.5) | 0.042 c |
|  | PWB | 49.7 (24.1) | 50.2 (27.8) | 55.6 (24.4) | 0.712 |
|  | RFr | 58.6 (25.6) | 54 (25.2) | 59.6 (28.2) | 0.637 |
|  | SPT | 62.6 (17.8) | 57.4 (23.4) | 70.8 (23.2) | 0.054 |
|  | RFa | 59.5 (30.5) | 70.5 (29.9) | 73.5 (31.3) | 0.088 |
|  | RHSC | 52.3 (28.4) | 46.3 (27.2) | 49.6 (30) | 0.701 |
|  | SSL | 48.3 (34.4) | 54.7 (28.9) | 59.4 (32.6) | 0.374 |
|  | COP | 47.6 (26.8) | 54.6 (28.3) | 56 (32) | 0.461 |
|  | REJ | 69.4 (27.8) | 72.9 (26.9) | 83.8 (25) | 0.055 |
|  | Total | 55.5 (16.6) | 55.9 (16.9) | 65.1 (20.3) | 0.146 |

* Kruskal-Wallis test (due to non-normality of distribution) + post-hoc analysis (Dunn’s test).

a > 2000 PLN > 1001-2000 PLN > 2000 PLN > (<1000 PLN);

b 1001-2000 PLN > (>2000 PLN);

c >2000 PLN > 1001-2000 PLN.

M, mean; SD, standard deviation; N, number of participants; p, statistical significance; ADL, activities of daily living; IADL, Instrumental Activities of Daily Living questionnaire; BDI, Beck Depression Inventory; EDSS, Expanded Disability Status Scale; PWB, psychological well-being; RFr, relationships with friends; SPT, symptoms; RFa, relationships with family; RHCS, relationship with the healthcare system; SSL, sentimental and sexual life; COP, coping with the disease; REJ, rejection.

**Supplementary Table 6.** Comparison of questionnaire results by duration of illness.

|  | | < 5 years (*n*=44) | 5-10 years (*n*=21) | > 10 years (*n*=35) | *p* * |
| --- | --- | --- | --- | --- | --- |
|  |  | *M (SD)* | *M (SD)* | *M (SD)* |  |
| ADL | | 4.7 (2.1) | 5 (0.9) | 4.5 (1.8) | 0.356 |
| IADL | | 23.1 (1.6) | 20.4 (2.8) | 20.3 (3.7) | <0.001 a |
| BDI | | 17.9 (12.1) | 18.1 (12.3) | 15.2 (7.6) | 0.729 |
| EDSS | | 1.4 (1.4) | 3.8 (2.1) | 4.9 (2) | <0.001 b |
| MusiQOL | ADL | 71.7 (18.8) | 47.8 (21.9) | 43.7 (21.9) | <0.001 a |
|  | PWB | 48.9 (27.8) | 51.5 (29.1) | 54.8 (19.6) | 0.547 |
|  | RFr | 56.9 (26.1) | 57.9 (26) | 57.1 (26.6) | 0.987 |
|  | SPT | 64.5 (22.5) | 62.6 (24) | 61.4 (20.1) | 0.783 |
|  | RFa | 67.6 (32.3) | 73.4 (28.1) | 63.1 (30.7) | 0.386 |
|  | RHSC | 51.4 (28.6) | 50.4 (27.2) | 46.5 (29.1) | 0.704 |
|  | SSL | 51.1 (36.5) | 62.5 (27.4) | 51.2 (28.1) | 0.342 |
|  | COP | 46.8 (31.2) | 53.6 (31.9) | 58.6 (22.4) | 0.162 |
|  | REJ | 78.3 (26.5) | 58.3 (34.5) | 80 (18.2) | 0.044 a |
|  | Total | 60 (19.1) | 57.6 (21.6) | 56.6 (14.2) | 0.823 |

* Kruskal-Wallis test (due to non-normality of distribution) + post-hoc analysis (Dunn’s test).

a < 5 years > (> 10 years), < 5 years > 5-10 years;

b >10 years > (< 5 years), 5-10 years > (< 5 years).

M, mean; SD, standard deviation; N, number of participants; p, statistical significance; ADL, activities of daily living; IADL, Instrumental Activities of Daily Living questionnaire; BDI, Beck Depression Inventory; EDSS, Expanded Disability Status Scale; PWB, psychological well-being; RFr, relationships with friends; SPT, symptoms; RFa, relationships with family; RHCS, relationship with the healthcare system; SSL, sentimental and sexual life; COP, coping with the disease; REJ, rejection.

**Supplementary Table 7.** Presentation of p value for the correlation analysis.

|  | 1 | 2 | 3 | 4 | 5 | 6 | 7 | 8 | 9 | 10 | 11 | 12 | 13 |
| --- | --- | --- | --- | --- | --- | --- | --- | --- | --- | --- | --- | --- | --- |
| 1. ADL | - |  |  |  |  |  |  |  |  |  |  |  |  |
| 2. IADL | **0.027** | - |  |  |  |  |  |  |  |  |  |  |  |
| 3. BDI | **0.037** | **0.036** | - |  |  |  |  |  |  |  |  |  |  |
| 4. EDSS | >0.05 | **<0.001** |  | - |  |  |  |  |  |  |  |  |  |
| 5. ADL | **0.008** | **<0.001** | **0.001** | **<0.001** | - |  |  |  |  |  |  |  |  |
| 6. PWB | >0.05 | >0.05 | **<0.001** | >0.05 | **0.003** | - |  |  |  |  |  |  |  |
| 7. RFR | >0.05 | >0.05 | **<0.001** | >0.05 | >0.05 | **<0.001** | - |  |  |  |  |  |  |
| 8. SPT | >0.05 | **0.018** | >0.05 | >0.05 | **<0.001** | **<0.001** | **0.002** | - |  |  |  |  |  |
| 9. RFA | **0.045** | **0.034** | >0.05 | >0.05 | **0.026** | **<0.001** | **<0.001** | **<0.001** | - |  |  |  |  |
| 10. RHSC | >0.05 | >0.05 | **0.002** | **0.039** | >0.05 | >0.05 | **<0.001** | >0.05 | **<0.001** | - |  |  |  |
| 11. SSL | **0.041** | >0.05 | **<0.001** | >0.05 | **0.039** | **<0.001** | **<0.001** | **<0.001** | **<0.001** | >0.05 | - |  |  |
| 12. COP | >0.05 | >0.05 | **<0.001** | >0.05 | >0.05 | **<0.001** | **0.022** | **0.015** | **0.013** | >0.05 | **0.003** | - |  |
| 13. REJ | >0.05 | **0.002** | **<0.001** | >0.05 | **<0.001** | **<0.001** | **0.001** | **0.001** | **<0.001** | >0.05 | **0.006** | **<0.001** | - |
| 14. MusiQOL - total | >0.05 | **0.003** | **<0.001** | **0.037** | **<0.001** | **<0.001** | **<0.001** | **<0.001** | **<0.001** | **<0.001** | **<0.001** | **<0.001** | **<0.001** |

ADL, activities of daily living; IADL, Instrumental Activities of Daily Living questionnaire; BDI, Beck Depression Inventory; EDSS, Expanded Disability Status Scale; PWB, psychological well-being; RFr, relationships with friends; SPT, symptoms; RFa, relationships with family; RHCS, relationship with the healthcare system; SSL, sentimental and sexual life; COP, coping with the disease; REJ, rejection.
